# Integrated vegetation management within electrical transmission landscapes promotes floral resource and flower-visiting insect diversity

**DOI:** 10.1371/journal.pone.0308263

**Published:** 2024-08-21

**Authors:** Chase B. Kimmel, Ivone de Bem Oliveira, Joshua W. Campbell, Emily Khazan, Jonathan S. Bremer, Kristin Rossetti, Matthew Standridge, Tyler J. Shaw, Samm Epstein, Alexandra Tsalickis, Jaret C. Daniels

**Affiliations:** 1 McGuire Center for Lepidoptera and Biodiversity, Florida Museum of Natural History, University of Florida, Gainesville, Florida, United States of America; 2 United States Department of Agriculture Agricultural Research Service Northern Plains Agricultural Research Laboratory, Sidney, Montana, United States of America; 3 Florida Department of Agriculture and Consumer Services, Division of Plant Industry, Entomology Section, Gainesville, Florida, United States of America; 4 Department of Geosciences, Auburn University, Auburn, Alabama, United States of America; 5 Department of Entomology and Nematology, University of Florida, Gainesville, Florida, United States of America; Southeastern Louisiana University, UNITED STATES OF AMERICA

## Abstract

Electrical transmission rights-of-way are ubiquitous and critical infrastructure across the landscape. Active vegetation management of these rights-of-way, a necessity to deliver electricity more safely, maintains these landscape features as stages of early successional habitat, a rarity in many regions, making these areas viable movement corridors for many taxa. The goals of this study were to (i) evaluate the effects of different electrical transmission landscape management practices on flowering plant and flower-visiting insect diversity parameters and (ii) generate conservation management inferences for these landscapes. In this study we tested the impact of three vegetation management levels across 18 electrical transmission sites. We evaluated the effects of treatment on bloom abundance and species richness as well as flower-visiting insect abundance and family richness. We identified 76541 flowers/inflorescences across 456 transects, including 188 species in 56 plant families. Additionally, we obtained data on 11361 flower-visitoring insects representing 33 families from 2376 pan trap sets. High vegetation management favored the reduction of coarse woody debris in the sites and harbored the highest level of abundance and richness of both floral resources and flower-visiting insects. We discuss that we can align social and ecological values of rights-of-way, ensuring their sustainability by applying regular and targeted integrated vegetation management. Thus, we can use rights-of-way landscapes not only as an effective management strategy for the delivery of essential human services, but also to provide conservation benefits for wild pollinators.

## Introduction

Management in human-dominated landscapes has led to the reduction in many types of native land cover. Since the mid-to late twentieth century, land management practices have focused on encouraging forest regeneration [1,2], including forest generation in areas with naturally heterogeneous land cover [3]. These vegetation management practices which often strive to minimize disturbance (e.g., fire suppression, flood prevention), limit the spatial coverage and inhibit generation of naturally occurring early successional habitats, ultimately reducing landscape heterogeneity [1]. This management style continues despite decades of documentation of the importance of early successional habitats for biodiversity (including habitat specialists), food web dynamics, and other ecological processes [3,4].

Flower-visiting insects, many of which act as plant pollinators, benefit from early successional habitat in a landscape [5,6]. While early successional habitat can be defined in a variety of ways, here we define it as being characterized by a mix of annual and perennial forbs, grasses, scattered shrubs, and an open canopy. The open canopy and low basal area characterizing these habitats provide ideal conditions for herbaceous flowering plants whose nectar and pollen resources attract visitors across seasons. Particularly considering widespread insect declines [7,8], sustainable management plans should consider aligning social needs (i.e., safe and efficient electrical service distribution) with promoting habitats critical to supporting the conservation of native insect populations.

Electrical transmission rights-of-way (ROW), also known as electric power transmission ROW or powerline ROW, must be managed to safely deliver electricity. This typically involves maintaining the ROW as an early successional habitat, minimizing woody vegetation. Even though ROW have been studied within the context of insect conservation and are well documented as being beneficial for many taxa, including plants [9], insect pollinators [10,11], birds [12], and small mammals [13], this landscape still has received relatively little attention [9].

Electrical transmission ROW tend to be linear and span large geographic scales, and are estimated to be larger than the area of almost any national park [14]. Active ROW management—that is removing and preventing growth of woody vegetation—often involves applying selective herbicide and/or mechanical clearing. This combination of management tools is often referred to as Integrated Vegetation Management (IVM), which allows for easier, safer access to electric lines for repairs, improves transmission reliability, reduces long-term vegetation management costs, and ensures safety for the habitat and energy consumer [15]. A byproduct of having less woody encroachment is delivery of more light to the ground and herbaceous layers, providing habitat for early successional species. In addition, the linearity and distance covered by ROW make these areas viable movement corridors for native bees and other widlife [16].

Beneficiaries of these movement corridors include early successional habitat-associated species such as many insect pollinators [14,17,18]. The ubiquity of electrical transmission ROW, the replication across the landscape, and the management practices used in this landscape, make ROW ideal study systems for investigating the ecology of various stages of early successional habitats and their use by insect pollinators. To our knowledge, there are no studies evaluating the effect of different management intensities in ROW on plants and flower-visitor communities in landscapes representative of the United States southern coastal plain. Addressing such pressing issues would contribute to better understanding the landscape created in ROW and encourage sustainable management solutions that can promote both viability for electrical service distribution and pollinator habitat.

Here we investigate the effect of ROW management on the plant and insect communities therein across a large geographic scale representative of the broader landscape of the United States southern coastal plain. We quantified and compared resource availability (e.g., flower abundance) and the community of flower-visiting insects across three electrical transmission ROW management intensities. Management ranged on a gradient from low intensity, or limited management having dense and high woody vegetation, to high intensity, with active removal and treatment of woody vegetation with regular mowing and/or herbicide. Thus, three management treatments were established: low, mid, and high according to intensity of woody plant removal. We predicted higher abundance and richness of flower-visiting insects and flowering plants in high intensity managed ROW. Our findings in this large landscape-scale study have implications for best practices for ROW management in similar regions and its potential of providing conservation benefits for wild pollinators while maintaining safe electrical transmission. These inferences can be applied to other systems requiring integrated vegetation management techniques.

## Methods

### Study sites and sampling design

Our study comprised 18 sites across Duke Energy managed electrical transmission grids in north-central Florida. Duke Energy provides electric utilities to serve 8.2 million customers across six states. Management is performed by Duke Energy based on cost and labor intensity to secure safe electrical transmission. Normally, the high intensity management is the desired intensity as it is the most cost-effective. Nevertheless, sites managed by Duke Energy present a range of conditions based on the management schedule, and some of the sites were behind their maintenance schedule. It should be noted that high intensity management activities on average only occurred once every two to three years.

**Fig 1:**
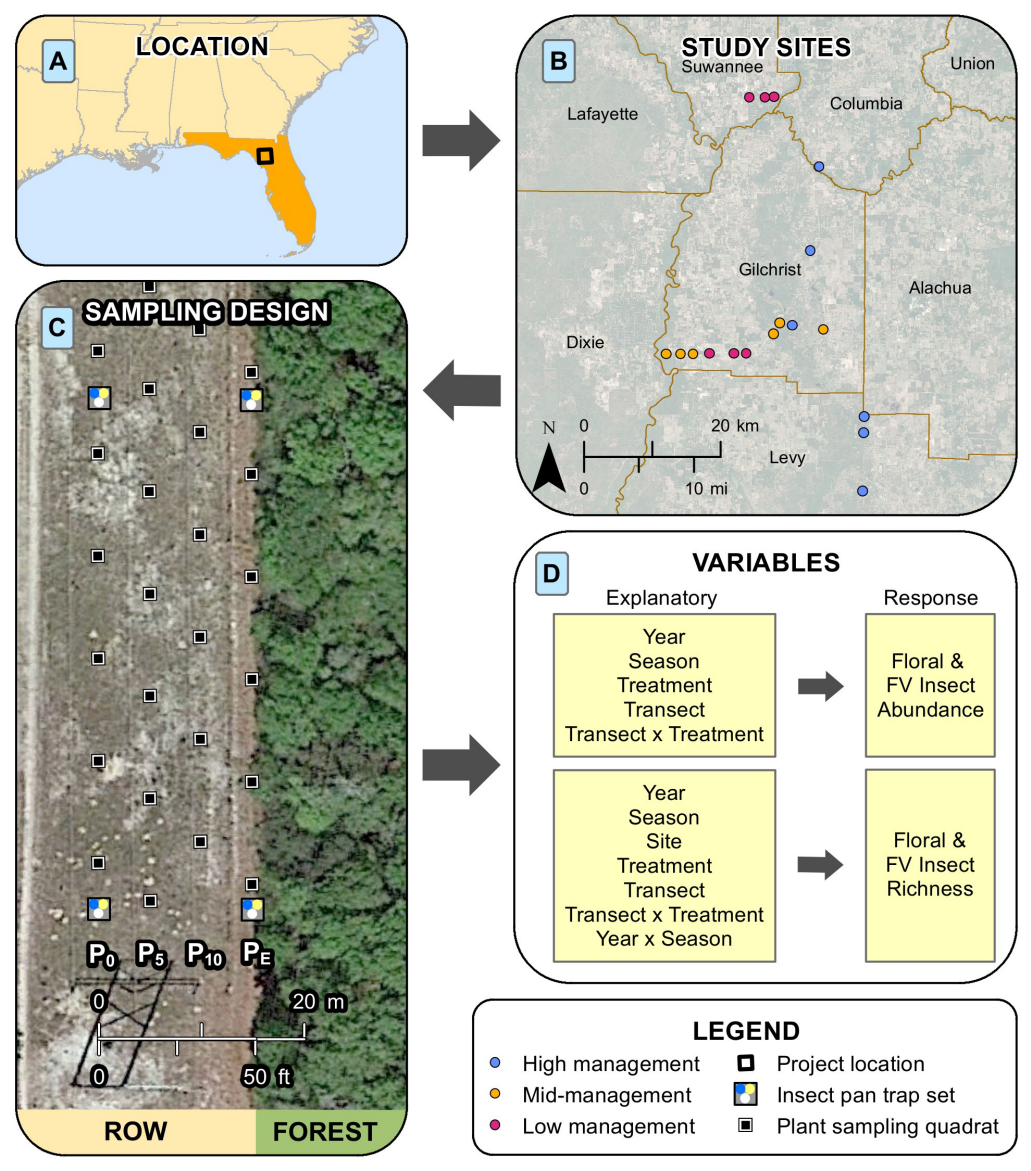
Study workflow. A) geographic location of project area, B) study site locations, C) demonstrative section of floral sampling and flower-visitor sampling design*, D) explanatory variables used in the model for the corresponding response variables. *Both plant and insect sampling transects extend beyond the extent shown in the figure.

We worked in collaboration with Duke Energy personnel to ensure access to sites that presented different levels of management. Duke Energy approved field site access and no permits for collection were required. Most sites occurred within Gilchrist County, with additional sites in Columbia, Levy, and Suwannee Counties. We assessed percentage of bare ground (BG) and percentage of woody debris (WD) for the different sites. This allowed us to categorize them into three management intensities (low, mid, and high; S1 Fig). The final experiment was comprised of six replicates per intensity management treatment, with higher intensity managed sites having more bare ground and less woody debris (average WD was 93%, 70%, and 54%, average BG was 2%, 7%, and 27%, respectively from low, mid, and high management intensities; S1 Fig). Each site was bordered on at least one side by upland mixed forest. At each site, transects were established for both floral and insect sampling (Fig 1).

### Floral sampling

To measure floral abundance and richness within the ROW, we conducted floral sampling surveys in four seasons (i.e., spring, early-summer, late-summer, and fall) for two years (i.e., 2017 and 2018). Each season when floral sampling occurred, all 18 sites were surveyed within one week. We established four 200 m transects per site located directly under the electric powerline (P_0_), five meters from the powerline (P_5_), ten meters from the powerline (P_10_), and 15 meters from the powerline or the edge of the ROW (P_E_) (Fig 1). Using a 1 x 1 m vegetation composition sampling quadrat as a sampling unit, we identified and quantified bloom/inflorescence abundance for each blooming species (i.e., total floral abundance per species and not per individual plant), estimated percentage of bare ground, and recorded presence/absence of coarse woody debris (i.e., dead woody vegetation ≥ 6 mm in diameter). For blooms that were unable to be identified to species in the field, we took a photograph and/or voucher of the plant for identification in the lab. Along each 200 m transect, we placed a total of 20 quadrats 10 m apart. Initial quadrats were placed at a random location (between 0 and 9 meters) using a random number generator. To determine how well the plant community was sampled, rarefaction curves were generated using iNext package [19,20] and evaluated considering the variation in the extrapolated values. This analysis was based on 10000 random samplings.

### Insect sampling

To estimate flower-visiting insect abundance and richness, at each site we placed four sets of three pan (or bowl) traps along the center and edge of the ROW (i.e., P_0_ and P_E_). Each set contained a white, yellow, and blue pan trap, and sets were placed approximately 50 meters apart (Fig 1), creating a 150 m long transect. Pan traps have been found to be an efficient collecting method for ROW in the southeastern United States [21]. Pan traps were filled with water and a drop of soap and placed at the same height as the surrounding vegetation. Pan trap deployment took two days to access and set up at all sites. After 48 hours of passive sampling, we returned to collect the contents of each set of traps which were filtered using a 190 micron strainer. The filtrate was then preserved in individually labeled vials containing 95% ethyl-alcohol. Specimens were sorted based on known flower-visitor groups: bees (Hymenoptera: Apiformes), wasps (Hymenoptera: non-Apiformes), beetles (Coleoptera), butterflies and moths (Lepidoptera), and flies (Diptera). For this study, known flower-visitors were defined as adult insects that visit the reproductive features of the flower (i.e., stamen or pistil) to obtain either pollen or nectar. Each group was identified to the lowest taxonomic unit possible, but for the analysis, only family level resolution was included. Insect traps were deployed monthly from April 2017 to October 2018 except for December and January when most flower-visiting insects in the region are dormant. To determine how well the flower-visiting insect community as well as each insect group was sampled, rarefaction curves were generated using iNext package [19,20] and evaluated considering the variation in the extrapolated values. This analysis was based on 10000 random samplings.

### Data analysis

To evaluate the effect of ROW management intensities on the flowering plant and flower-visiting insect communities, abundance and richness data for plants and insects were analyzed. It should be noted that extrapolated values were not utilized for the analysis. Model inputs included treatment intensity (management level), year, season, and interactions between these factors. Blooming plant species abundance and richness was calculated as the total number of blooms per transect (i.e., per 20 m^2^) and the total number of flowering plant species observed per transect, respectively. Flower-visiting insect abundance was defined as the total number of flower-visiting insects captured per transect (i.e., from four sets of bowl traps). Flower-visiting insect family richness was the cumulative number of different flower-visiting insect families captured per transect.

### Model details

For all analyses involving abundance, the effect of ROW management was tested using both glm and zero inflated analyses. Goodness-of-fit was evaluated for the insertion of different effects in the model and Vuong test was performed to compare non-nested glm and zero-inflated models. Model and tests were implemented using the pscl package v.1.5.5 [22]. Given the high level of overdispersion observed in the data, zero inflated regressions had a better fit than glm models for all abundance analysis performed. The effects added in the model were chosen considering parsimony. The final model used in the analysis for abundance was:

$$\bar{y}=\mu +X_{1}ye+X_{2}se+X_{3}t+X_{4}ta+X_{5}t*ta+e$$ (1)

Where $\bar{y}$ is the vector for response variable (i.e., abundance), *μ* is overall mean, *ye* is the effect of year, *se* the effect of season, *t* the effect of ROW management treatments, *ta* the effect of transects, t*ta the effect of treatment by transect interaction, and *e* represents the vector for the random residual error. *X*_*1*_, to *X*_*5*_, are the incidence matrices for year, season, treatment, transect, and the interaction of treatment and transect, respectively.

For all analyses involving richness, the effect of ROW management was evaluated using the following generalized linear model:

$$\bar{y}=\mu +Xye+Z_{1}se+Z_{2}si+Z_{3}t+Z_{4}ta+Z_{5}ye*se+Z_{6}t*ta+e$$ (2)

In this model, $\bar{y}$ is the responsible variable (i.e., richness), *μ* is overall mean, *ye* is the effect of year, *se* the effect of season, *si* the effect of site, *t* the effect of ROW management treatments, *ta* the effect of transects, ye*se is the effect of year by season interaction, t*ta the effect of treatment by transect interaction, and *e* the vector for the random residual error. *X*, *Z*_*1*_ *to Z*_*6*_ are the incidence matrices for the effects of year, season, site, treatment, transect, the interaction of year and season, and the interaction of treatment and transect, respectively; only year was considered as a fixed effect. This model was implemented using the packages asreml.R v 4.1.0.11 [23].

A Poisson distribution was assumed for both models due to the nature of the data and its skewed distribution. To verify significance between the factors tested in each analysis, post hoc tests assuming Sidak correction for multiple comparisons were performed (σ = 0.05), using functions implemented in the package emmeans v. 1.7.5 [24]. Graphical visualizations were generated using ggplot2 [25] and gghalves v. 0.1.3 [26]. All analyses were completed in the R platform v. 4.2.0 [27].

## Results

### Floral and insect sampling

We identified 76541 flowers/inflorescences across 456 transects over the course of this project. This included 188 species in 56 plant families. The vast majority of blooming plants were native (163 native vs. 24 non-native) and three were endemic to Florida. In Gilchrist County, where most of the sites were located, there were 45 new county records based upon known occurrences from the Florida Plant Atlas [28]. When combining both years of data and looking at bloom phenology, the average number of blooming species increased from spring, early summer, late summer, and fall (35, 83, 88, and 94, respectively). A complete inventory, duration, growth habit, native and endemic status, as well as seasonal bloom phenology can be found in S1 Table. It should be understood that these numbers do not represent all plants at the site as non-blooming plants were not identified and many wind pollinated plants, such as sedges and grasses, were not included.

**Fig 2:**
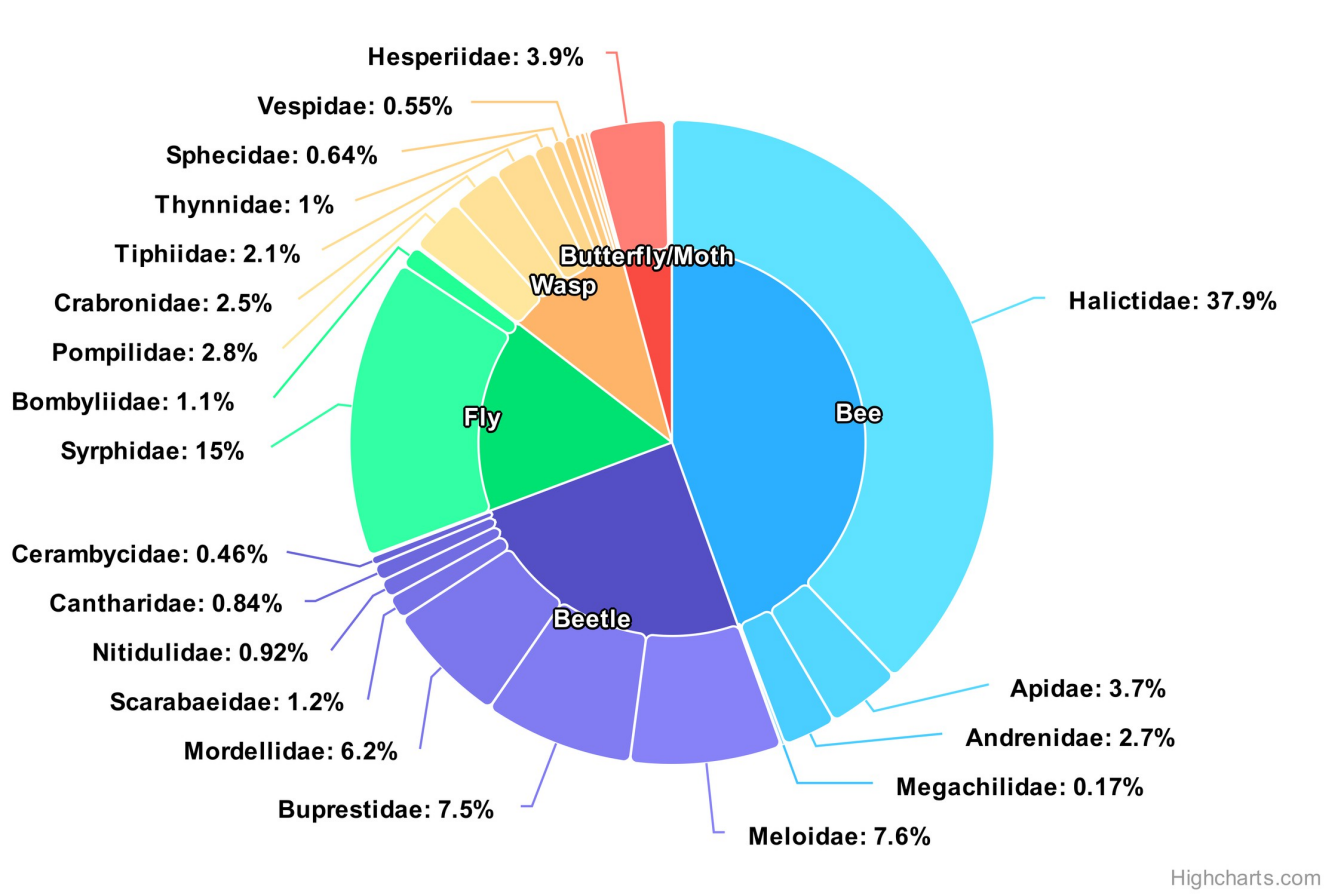
Piechart for insect families. Relative proportions of all insect families collected across all transect in all treatments.

In total, we collected 11361 flower-visitors representing 33 families from 2376 pan trap sets distributed across 606 transects. The most collected insect group was bees, followed by beetles, flies, wasps, and butterflies/moths (44.5%, 24.8%, 16.2%, 10.3%, and 4.2%, respectively). The most common family observed was Halictidae (Hymenoptera) with 4303 individuals or 37.9% of the total number of insects collected (Fig 2).

Rarefaction curves for plant species richness and insect family richness for each treatment can be found in S2 Fig, where a relatively low confidence interval for the extrapolated values was estimated. Rarefaction curves for insect family richness for each insect group can be found in S3 Fig. Both butterfly/moth and flies had more variation in their extrapolated values. Nevertheless, our rarefaction curve results indicate that sampling was effective for all other insect taxa and plants.

### Floral abundance and species richness

**Fig 3:**
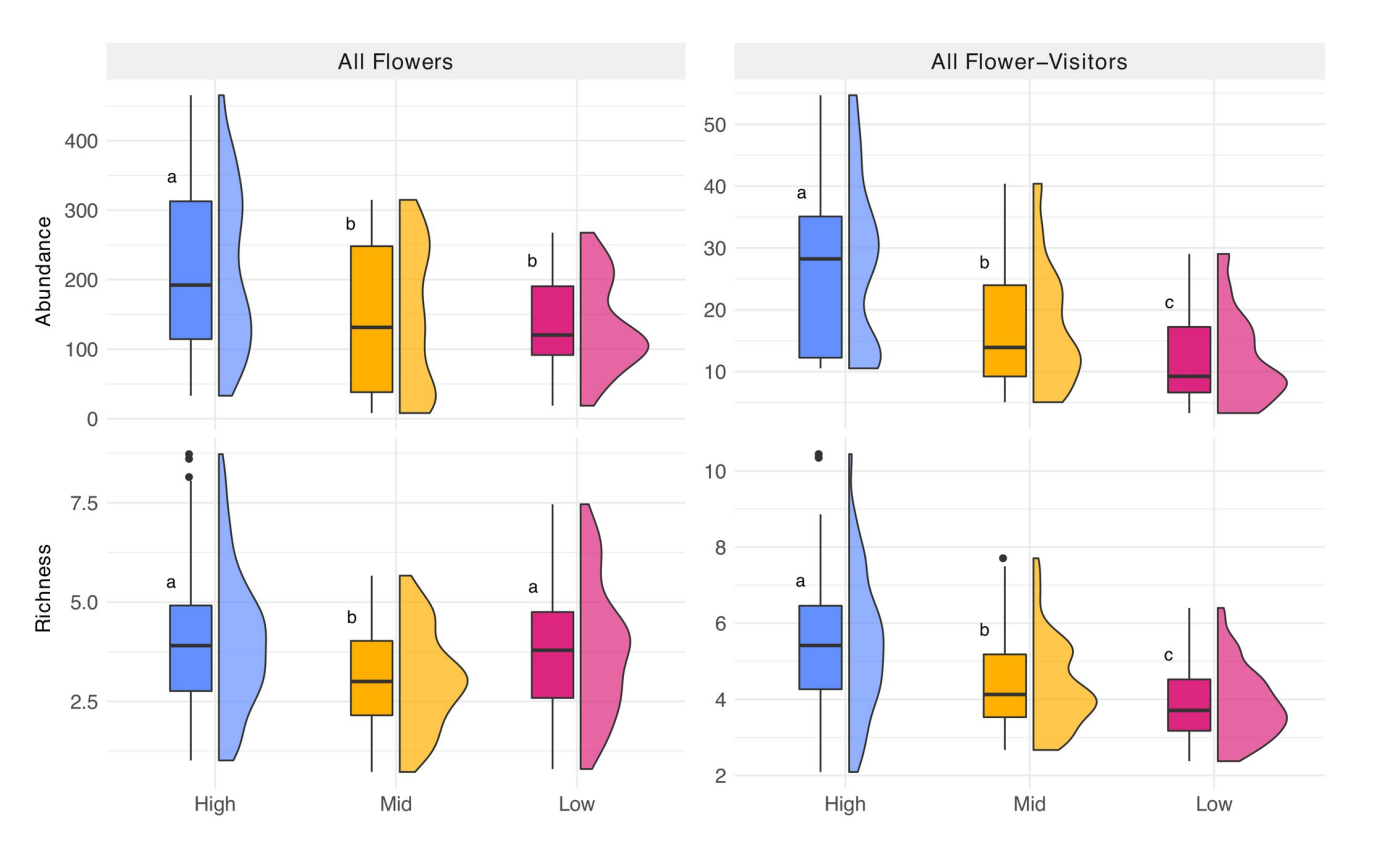
Flower and flower-visitor abundance and richness. Boxplot and half-violin plots of all flowering plant abundance and species richness and flower-visitor abundance and family richness for each corresponding management treatment. Letters based on post hoc tests assuming Sidak correction for multiple comparisons. Confidence level used: 0.95, significance level used: σ = 0.05 (a>b>c).

Electrical transmission ROW management intensities significantly impacted floral abundance and species richness. Both parameters presented the highest averages in the transects with the highest degree of management. Floral abundance decreased as management level decreased, with lowest average floral abundance found in low intensity managed sites, that is sites with a high percentage of woody debris and lowest percentages of bare ground (i.e., 214.75, 147.41, and 134.22 for high, mid, and low management intensities, respectively; Fig 3; S2 Table). However, floral abundance observed for mid and low management intensities, did not differ significantly. While average species richness of flowers in high intensity managed ROW was significantly higher than mid or low intensity managed ROW (i.e., 4.02, 3.07, 3.82, respectively; S2 Table), only the mid-managed intensity ROW species richness was significantly lower than the other two treatments (Fig 3).

### All flower-visitors insect abundance and family richness

Overall, we found the highest abundance of flower-visiting insects in sites with high management intensities and significantly less abundances as management intensity decreased (means = 27.53, 17.12, 11.91 for high, mid, and low management intensities, respectively; Fig 3; S2 Table).

Flower-visiting insect family richness followed the same pattern as flower-visiting insect abundance (Fig 3). The highest family richness of flower-visiting insects was associated with high management intensity and family richness averages were significantly smaller as management level decreased (means = 5.42, 4.37, 3.88 for high, mid, and low management intensities, respectively; Fig 3; S2 Table).

### Flower-vising insect group abundance and family richness

**Fig 4:**
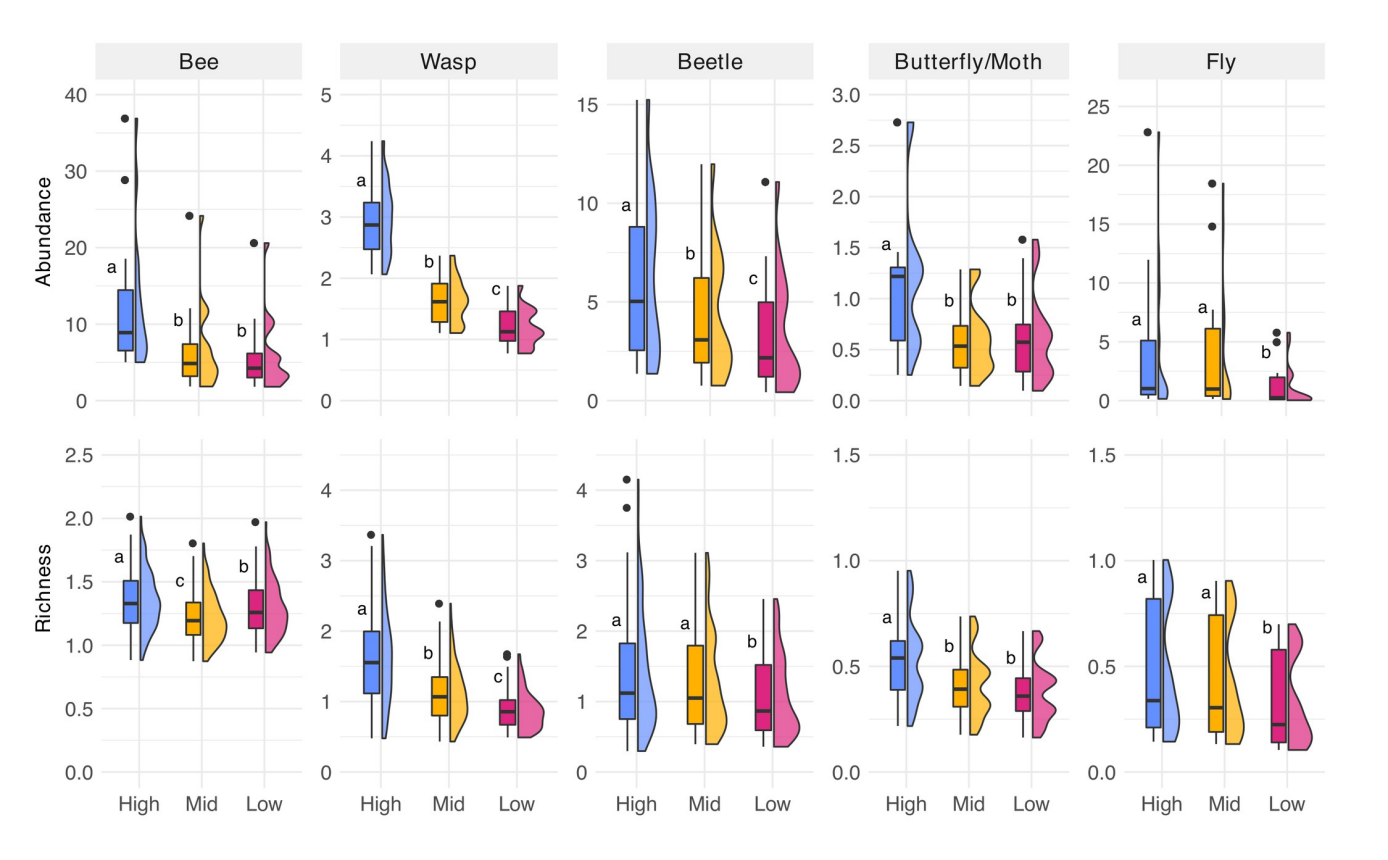
Flower-visitor abundance and richness for each insect group. Boxplot and half-violin plots of flower-vising insect abundance and family richness for each insect group within corresponding management treatment. Letters based on post hoc tests assuming Sidak correction for multiple comparisons. Confidence level used: 0.95, significance level used: σ = 0.05 (a>b>c).

The pattern of decreasing insect abundance with decreasing management held across most individual insect groups (Fig 4; S3 Table). The lowest mean family abundance values were observed for butterfly/moths (0.59) on low management intensity, while the highest mean abundance level was observed for bees (12.56) on high management intensity (S3 Table). All insect groups, with the exception of flies, had significantly higher abundances in high management intensity sites than mid and low management intensity sites (Fig 4).

There were significantly more families of bees, wasps, and butterfly/moths in high management intensity sites than in mid and low management intensity sites (Fig 4). Nevertheless, for beetles and flies, mean richness for high and mid management intensity did not significantly differ from each other. The highest mean richness was observed for wasps in high management intensity treatment (1.60), and flies in low management intensity treatment presented the lowest mean richness (0.32). Associated statistical results can be found in S3 Table.

## Discussion

In this study, we assessed how the management intensity of electrical transmission rights-of-way (ROW) landscapes impacted floral resources and flower-visiting insect diversity through their overall abundance and richness metrics. For this, we tested the impact of three vegetation management levels across 18 sites in a large habitat representative of the southern coastal plain in the United States. We considered measurements from an extensive dataset of flowering plants and flower-visitor communities to generate information that can mutually support positive outcomes for the transmission of energy and for insect conservation.

Landscape composition can be largely determined by human activities. The correct management within anthropogenic systems can align social needs while generating habitat suitable for a vast range of insects, acting as biological reserves [29–31]. Here we show that management affected the abundance and richness levels not only of flowering plant species but also positively influenced insect flower-visitors across multiple taxonomic groups. Besides, confirming what was found in the literature about the influence of ROW vegetation management on flower-visiting insects [14,31,32], we were able to study a section of the United States for which, to our knowledge, there were no studies yet developed. Our study also brings the distinction of evaluating multiple insect taxa groups, while most studies focused on a specific taxonomic group [9–13].

Our results show that active and regular management of primarily woody vegetation within this landscape increased abundance and richness of flower-visiting insects. Patterns of insect abundance and richness are in parity with the patterns observed for flowering plants; with increasing management the abundance of flowers increased, providing subsidies for insects which rely on and are attracted to flowers. It is interesting to notice that even though low intensity management has presented similar levels of flowering plant species richness as the high intensity management, the abundance of flowering plants was significantly higher on high intensity management. The higher number of flowers with high intensity management may have contributed to the results observed for flower-visitors richness and abundance, which were also significantly higher in high intensity management.

Active and regular management of electrical transmission ROW also resulted in a decrease of woody material, which, in turn, provided more habitat (i.e. bare ground, higher solar radiation) for early successional flowering plants to thrive. Similar results were observed by Eldegard [9], Wagner [32], and Burt and Rice [33] when comparing understory vegetation and forests adjacent to these clearing sites, showing that the reduction of woody debris and denser canopy can help increase diversity and abundance of flowering plants. Dense shrub layers beneath forest canopies that increase woody vegetation can negatively impact herbaceous plant cover and diversity, as well negatively affect pollinators [34]. These patterns held throughout the broad temporal and geographic scope of our study and across the taxonomic breadth of flower-visiting insects evaluated. These results confirm that successful implementation of landscape management within ROW can be invaluable, allowing the creation of millions of hectares of corridor-like habitats for pollinators [11,31], providing mutually supportive outcomes for people and nature. However, more studies should be developed to optimize ROW management focusing not only on human but also on biodiversity needs, given that the pressures exerted on these constructed environments can present significant challenges for the biodiversity they support. These stressors can include multiple parties managing the ROW (e.g., landowner, electric transmission providers, and other utility providers), invasive species, as well as impacts of being adjacent to agriculture and roadsides (e.g., spray drift, pollutants—such as heavy metals, de-icing materials, vehicle exhaust—and vehicle runoff) [35,36].

Rights-of-way can be classified as semi-natural habitats and can facilitate connectivity on multiple scales within the landscape. On a local scale, connectivity within fragmented or increasingly urban/agricultural landscapes can potentially facilitate and promote organism movement and habitat connectivity, as well as provide pollination and biocontrol services to nearby areas. Semi-natural habitats surrounding agricultural landscapes have shown increasing positive benefits such as increasing biodiversity and providing ecosystem services to agricultural landscapes [37–39]. This effect could be higher on crops dependent on insect pollinators. Even though flowering crops normally exceed the resources provided by wild plants when blooming, the flowering resources are limited in time [39]. Thus, the presence of complementary habitats with high diversity of blooming plants could be particularly beneficial to pollinator-dependent crops, as well as adjacent urban and natural systems. Besides revealing a significant increase in flower abundance with the implementation of high intensity management practices (Fig 3), our study also presents a comprehensive compilation of plant phenology data (S1 Table), underscoring the diverse range of flower phenologies within the ROW areas under examination. Thus, we show evidence of positive impact of management on floral metrics and highlight the rich phenological diversity of blooming plants in ROW environments. Electrical transmission ROW can also harbor rare plants or animals [11,16,40–43]. Three state endemic plant species were observed, of which two of them require full sun to minimal shade [44,45]. Thus, management to create more open, sunny locations can benefit these species. Many of the observed forbs and herbs listed in S1 Table grow optimally when given full sun, thus overshading due to minimal management could negatively impact many species.

On a larger scale, the corridor-like aspect of ROW could also play an important role for insects that require continental migration, such as monarch butterflies (*Danaus plexippus*). Migratory insects require high energy food sources distributed throughout their migratory path. Habitat loss and fragmentation are among the main factors that have significantly impacted the available resources for conservation of biological diversity [46–48] and have a high effect on migratory species [49–51]. Linear elements within fragmented landscapes, such as ROW, could supply the resources needed for migratory species if correctly managed by increasing connectivity. For example, the presence of *Asclepias tuberosa*, a known nectar source and host plant for the monarch butterfly, was found within the sites evaluated, as well as other flowers that are known nectar resources for monarchs and other butterflies (S1 Table). Thus, our results also confirm that ROW could be a target for local conservation efforts. While our study did not investigate connectivity, this environment could play an essential role as biological corridors for migrating insects, given that management increased abundance and richness of flowering plants. Further studies on the effect of ROW management practices on migratory insects could confirm this importance.

Management practices which limit encroachment of woody plants, and therefore maintain an early successional habitat, provide the necessary conditions for many species of native flowering herbaceous plants [9,34,52,53]. The patterns of insect abundance and richness increasing with management intensity demonstrate the lack of negative effects of integrated vegetation management, which can include selective herbicide use, manual clearing of woody plants, and occasional mowing/brush cutting. Indeed, this active management resulted in increased abundance of blooms, which attract and provide resources for many groups of flower-visiting insects. Net positive effects on insect abundance and richness held across insect groups, including many economically important pollinator groups (i.e., bees) and possible biological control agents like wasps.

When considering flower-visiting insects overall, increased management resulted in higher abundance and family richness. Nevertheless, we found partial congruence when analyzing responses of individual taxonomic groups to the different management practices tested. Across all groups we found higher abundances in highly managed ROW compared with ones receiving less frequent and intensive management, a trend also documented in similar studies (e.g., [11,40]), and those comparing ROW with forested sites (e.g., [10,16]). This trend was most apparent in wasps, a group that includes many predators and parasitoids, which likely benefited from the higher prey abundance in these ROW transects. The same trend was observed for the insect groups most reliant on flowers (i.e. bees, beetles, butterflies, and moths). All showed strong responses to management in parity with bloom abundance. That is, insects associated with flowers and pollination services were positively impacted by the active management of ROW. Flies, on the other hand, were only less abundant in the lowest management regime, perhaps because they rely on a wider suite of foraging resources (e.g., honeydew, dead organisms, dung, etc.) compared with the other groups examined. Hence, flower abundance may only be one part of the driving force for flies. Both richness and abundance of flies demonstrated a response to treatment with lower values in the least managed ROW. Therefore, these differences in abundance and richness, likely stem from the discrete behaviors, diets, and life histories of specific insect groups. It is also important to highlight that flower-visitor composition can be affected by the surrounding land use [38,54]. A more in-depth study evaluating surrounding land use of ROW could help in clarifying such interaction. Future analysis including beta-diversity and a lower taxonomic resolution can be performed to verify differences in community composition between treatments. Additionally, more analysis can be done to verify if different intensity management may have benefits for particular species.

It should be recognized that some of the differences observed on flower-visitor parameters could be due to the sampling bias of using only pan traps [55,56]. Contrary to our findings, some studies have found that capture rates of pan traps were lowest when flowering plant richness was highest [57]. Using only pan traps can lead to higher halictid counts [21,56,57], which was corroborated in our study. Nevertheless, collection biases exist with all collecting methods [58]. It is crucial to note that our analysis was conducted using the family level as the taxonomic resolution, where the impact of sampling bias is mitigated. Additionally, further studies could exploit other trapping methods, such as sweep netting, flight interception traps, and/or vane traps to capture additional flower-visiting groups that are not widely caught in pan traps [21]. Moreover, our rarefaction curve results indicate that sampling was effective (S1 Fig). However, when looking at the rarefaction curves for specific insect groups (S2 Fig), higher variation in the confidence interval for butterflies/moths as well as flies, show that additional sampling could benefit these groups. It should also be noted that many of the study sites were adjacent to roadways due to the nature of energy lines running parallel to roadways. As such, ROW adjacent to roadways could be impacted differently than ROW not adjacent to roadways.

Modern landscape management has the potential to reconcile the needs of humans as well as those of wildlife populations. Electrical transmission ROW are ubiquitous and expansive habitats which are ideally managed to limit woody encroachment. When actively managed to remove and prevent woody plants, ROW emulate natural patchy early successional habitat across the landscape, a habitat type lacking in modern times [31,59]. This management is also necessary for the safe and effective delivery of electricity to prevent disasters like wildfires [60] and to facilitate access for maintenance and repair. Thus, the practice of integrated vegetation management within the ROW offers benefits for conservation-minded land managers and electric companies alike. Moreover, besides the cost and time implicated on the removal of woody vegetation within the ROW, this practice can yield cost-effective advantages for electric companies by streamlining long-term management efforts.

Studies on how different management practices can impact flower and flower-visitor communities could complement the discoveries obtained in our study. Additionally, studying interactions between lower taxonomic resolutions, like flower-visitor species diversity, and ROW management can further guide plans addressing electrical transmission safety. This could generate a deeper understanding of how to establish management plans that can simultaneously address electrical transmission safety concerns while benefiting biological diversity and fostering conservation actions.

## Conclusions

Electrical transmission rights-of-way are a ubiquitous form of infrastructure. When actively managed, these long, linear strips across the landscape structurally and functionally resemble early successional habitat. These lands are typically under the jurisdiction of electric companies and are not normally managed by them as havens of biological diversity. Despite this, they harbor large amounts of floral resources, and native biodiversity including flower-visiting insects–a group with quantifiable benefits to ecosystem function, economic success, and pest control. Here we demonstrate that high management intensity of electrical transmission ROW has a significant positive impact on flowering plants and flower-visiting insect abundance and richness, mutually supporting positive outcomes for the transmission of energy and for pollinator conservation. Our results not only confirm the notion that actively managing electrical transmission ROW supports biodiversity, but also brings innovative information about abundance and richness for flowering plants and five flower-visitor taxa groups evaluated on these highly modified landscapes. This study is the first, to our knowledge, to evaluate the interaction of ROW management intensity with pollination related biodiversity on a habitat representative of the southern coastal plain in the United States. Given the geographic and taxonomic breadth examined in this study, the positive implications of integrated vegetation management for biodiversity and power safety are broadly applicable.

## Supporting information

S1 Fig Coarse woody debris and bare ground for each treatment. Site portions of coarse woody debris and bare ground indicating how treatments were assigned.
(TIF)

S2 Fig Plant and insect rarefaction curves. Interpolated and extrapolated individual-based rarefaction curves for plant species richness (A) and overall insect family richness (B) for each treatment.
(TIF)

S3 Fig Insect group rarefaction curves. Interpolated and extrapolated individual-based rarefaction curves for families of flower-visiting insect groups for each treatment. From left to right, bee (A), wasp (B), beetle (C), butterfly/moth (D), and fly (E).
(TIF)

S1 Table Blooming plant inventory and phenology. Detailed overview for plant inventory (S = Spring, ES = Early-Summer, LS = Late-Summer, and F = Fall) over the duration of the project. Duration and growth habit were determined using the USDA plant database [61]. Native and endemic status data was collected from the Atlas of Florida (AFP) plant database [28]. Potential new county records were determined using AFP. If the given species did not have a voucher listed for the county in which it was found, it was indicated as a potential new voucher record. In some situations, a given plant was unable to be identified to species. In these instances, each species within the respective genus was investigated to determine native, endemic, and potential new county record status. If the designation was consistent across all species, a designation was determined. However, if there were differences between different species within the genus, an NA was used to denote that no determination could be found. An asterisk indicates multiple varieties of this species are listed on AFP and specific county records could not be ascertained.
(PDF)

S2 Table Parameter summary table for plant and flower-visitors. Minimum, maximum, average (lsmean), standard error, and significance observed for abundance and richness for the analysis of all plant and all flower-visitors data across the three management treatments (i.e., high, mid, and low management). This table corresponds with Fig 2 in the manuscript. Letters indicating statistical differences based on post hoc tests assuming Sidak correction for multiple comparisons. Confidence level used: 0.95, significance level used: σ = 0.05 (a>b>c).
(PDF)

S3 Table Parameter summary table for flower-visitor insect groups. Minimum, maximum, average (lsmean), standard error, and significance observed for abundance and richness for five flower-visiting insect groups across the three management treatments (i.e., high, mid, and low management). This table corresponds to Fig 4 in the manuscript. Letters indicating statistical differences based on post hoc tests assuming Sidak correction for multiple comparisons. Confidence level used: 0.95, significance level used: σ = 0.05 (a>b>c).
(PDF)

S4 Table Blooming plant data. (XLSX)

S5 Table All flower-visitor data. (XLSX)

S6 Table Flower-visitor by group data. (XLSX)
