# Supplementary figures and images for: Integrated vegetation management within electrical transmission landscapes promotes floral resource and flower-visiting insect diversity
Source: PLoS One. 2024 Aug 21;19(8):e0308263. doi: 10.1371/journal.pone.0308263 (PMC11338444; doi:10.1371/journal.pone.0308263)

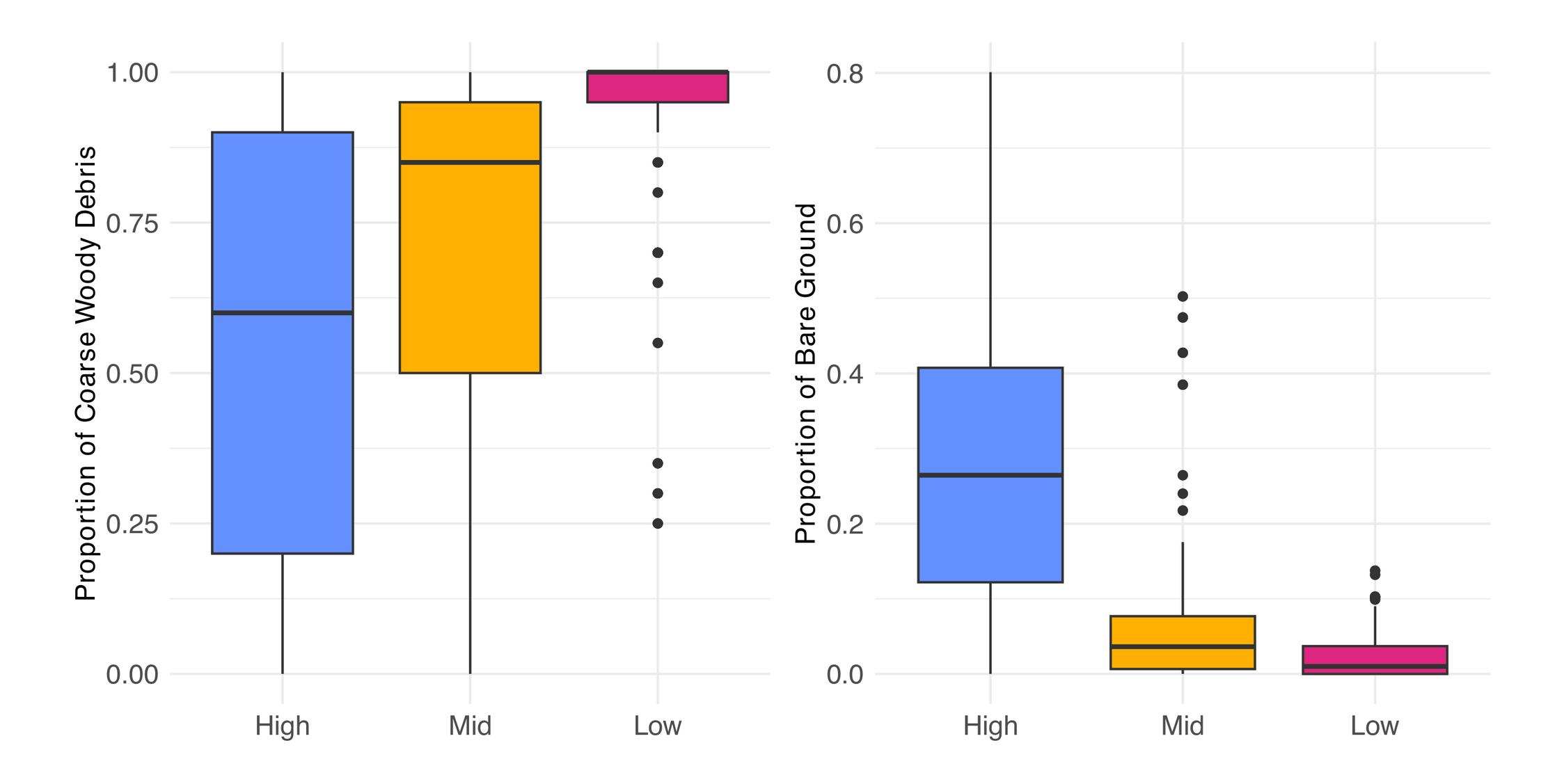

Supplement: S1 Fig — Site portions of coarse woody debris and bare ground indicating how treatments were assigned. (TIF) [file pone.0308263.s001.tif]

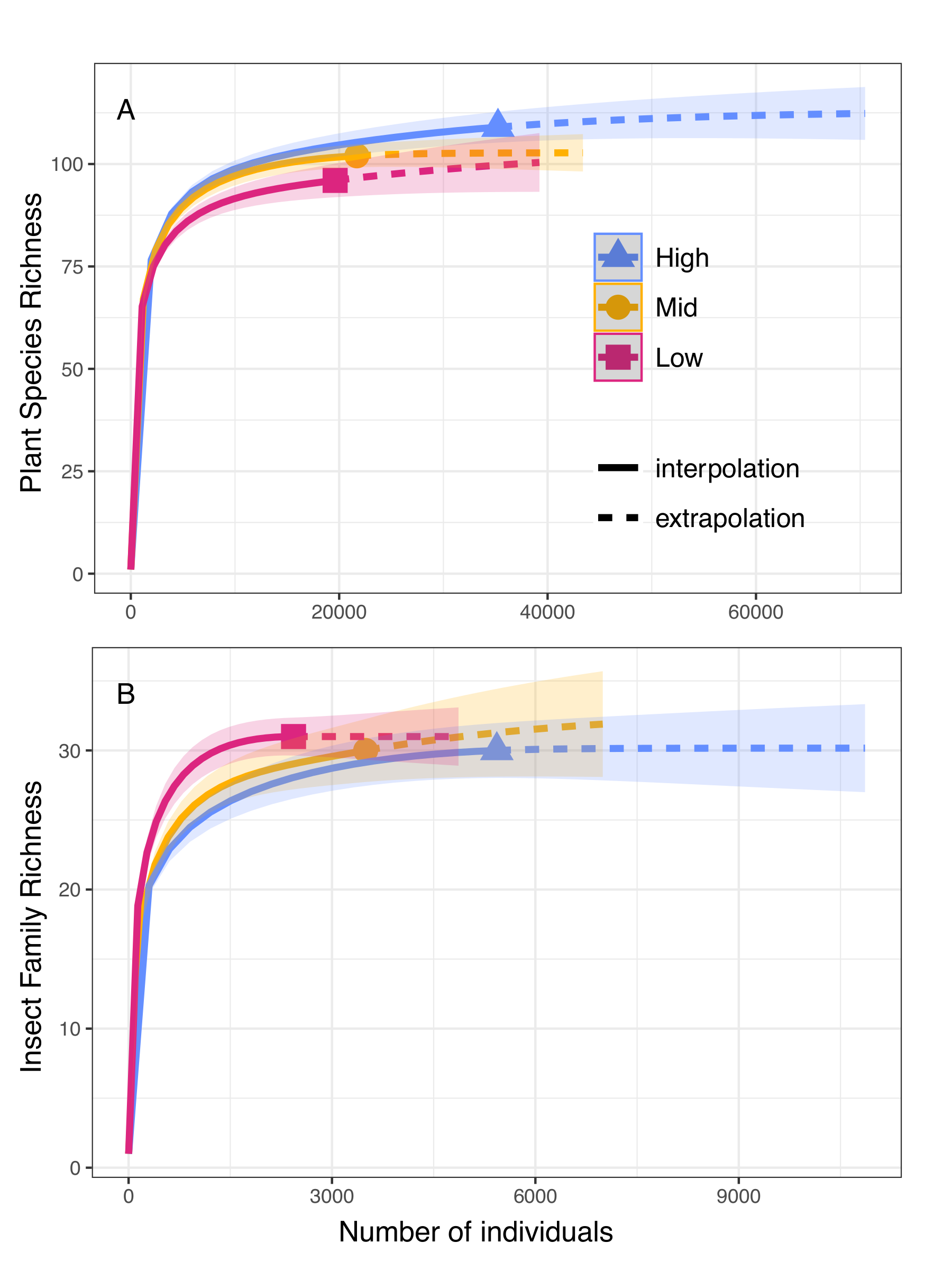

Supplement: S2 Fig — Interpolated and extrapolated individual-based rarefaction curves for plant species richness (A) and overall insect family richness (B) for each treatment. (TIF) [file pone.0308263.s002.tif]

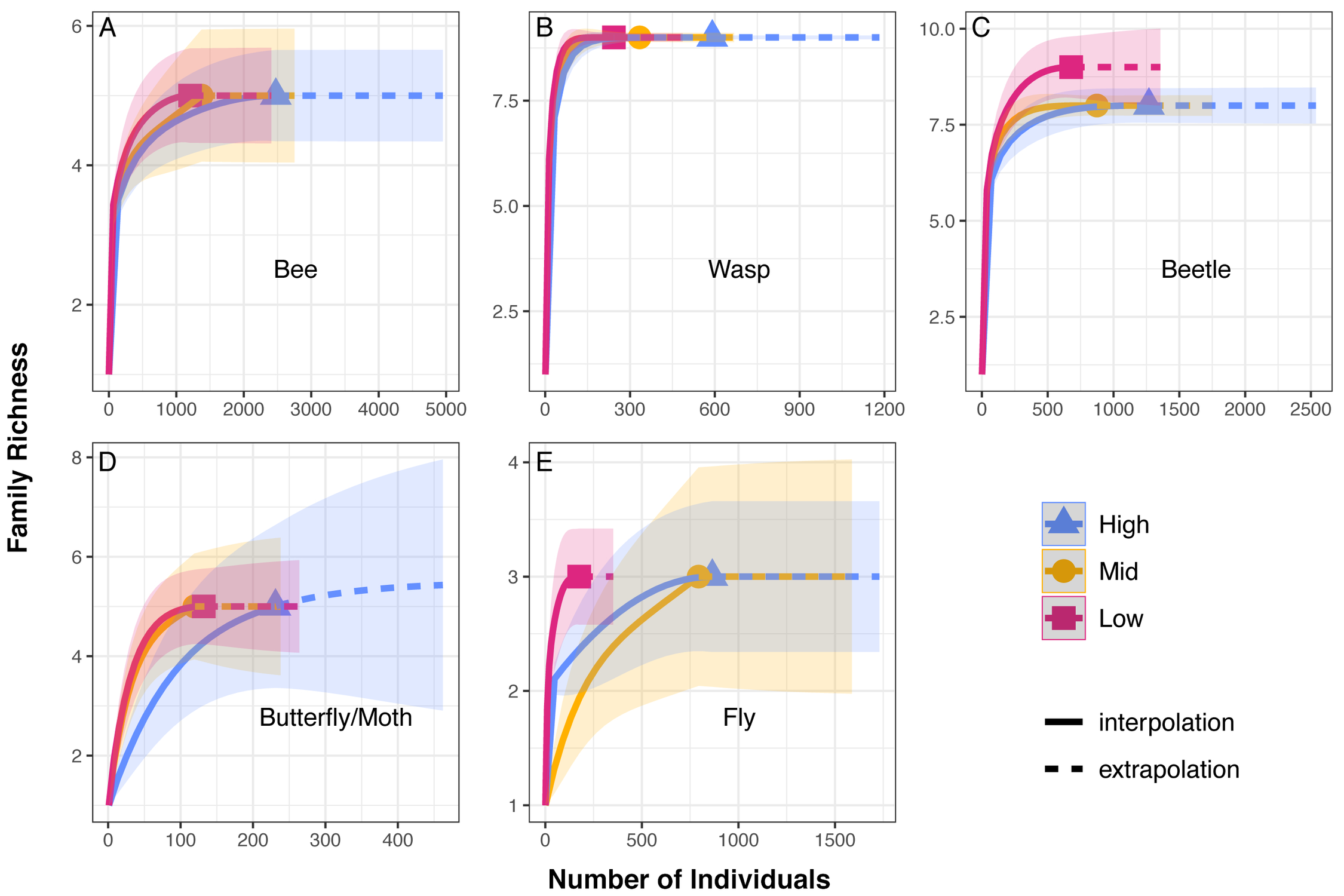

Supplement: S3 Fig — Interpolated and extrapolated individual-based rarefaction curves for families of flower-visiting insect groups for each treatment. From left to right, bee (A), wasp (B), beetle (C), butterfly/moth (D), and fly (E). (TIF) [file pone.0308263.s003.tif]
