# Supplementary material for: Integrated vegetation management within electrical transmission landscapes promotes floral resource and flower-visiting insect diversity
Source: PLoS One. 2024 Aug 21;19(8):e0308263. doi: 10.1371/journal.pone.0308263 (PMC11338444; doi:10.1371/journal.pone.0308263)
Supplement: S1 Table — Detailed overview for plant inventory (S = Spring, ES = Early-Summer, LS = Late-Summer, and F = Fall) over the duration of the project. Duration and growth habit were determined using the USDA plant database [61]. Native and endemic status data was collected from the Atlas of Florida (AFP) plant database [28]. Potential new county records were determined using AFP. If the given species did not have a voucher listed for the county in which it was found, it was indicated as a potential new voucher record. In some situations, a given plant was unable to be identified to species. In these instances, each species within the respective genus was investigated to determine native, endemic, and potential new county record status. If the designation was consistent across all species, a designation was determined. However, if there were differences between different species within the genus, an NA was used to denote that no determination could be found. An asterisk indicates multiple varieties of this species are listed on AFP and specific county records could not be ascertained. (PDF) [file pone.0308263.s004.pdf]

Integrated vegetation management within electrical transmission landscapes promotes floral resource and flower-visiting insect diversity

Chase B. Kimmel[1*], Ivone de Bem Oliveira[1], Joshua W. Campbell[1,2], Emily Khazan[1], Jonathan S. Bremer[1,3], Kristin Rossetti[1], Matthew Standridge[1], Tyler J. Shaw[1], Samm Epstein[1], Alexandra Tsalickis[1,4], and Jaret C. Daniels[1,5]

[1] McGuire Center for Lepidoptera and Biodiversity, Florida Museum of Natural History, University of Florida, Gainesville, Florida, United States of America

[2] United States Department of Agriculture Agricultural Research Service Northern Plains Agricultural Research Laboratory, Sidney, Montana, United States of America

[3] Florida Department of Agriculture and Consumer Services, Division of Plant Industry, Entomology Section, Gainesville, Florida, United States of America

[4] Department of Geosciences, Auburn University, Auburn, Alabama, United States of America

[5] Department of Entomology and Nematology, University of Florida, Gainesville, Florida, United States of America

* Corresponding author
E-mail: cbkimmel@ufl.edu (CBK)

S1 Table. Blooming plant inventory and phenology. Detailed overview for plant inventory (S = Spring, ES = Early-Summer, LS = Late-Summer, and F = Fall) over the duration of the project. Duration and growth habit were determined using the USDA plant database [61]. Native and endemic status data was collected from the Atlas of Florida (AFP) plant database [28]. Potential new county records were determined using AFP. If the given species did not have a voucher listed for the county in which it was found, it was indicated as a potential new voucher record. In some situations, a given plant was unable to be identified to species. In these instances, each species within the respective genus was investigated to determine native, endemic, and potential new county record status. If the designation was consistent across all species, a designation was determined. However, if there were differences between different species within the genus, an NA was used to denote that no determination could be found. An asterisk indicates multiple varieties of this species are listed on AFP and specific county records could not be ascertained.

							Bloom Phenology			
**Scientific Name**	**Family**	**Duration**	**Growth Habit**	**Native**	**Florida Endemic**	**New County Record**	**S**	**ES**	**LS**	**F**
Agalinis fasciculata	Orobanchaceae	Annual	Forb/herb	Y	N					X
Asclepias tuberosa	Apocynaceae	Perennial	Forb/herb	Y	N				X	
Asemeia violacea	Polygalaceae	Annual	Forb/herb	Y	N				X	X
Asimina angustifolia	Annonaceae	Perennial	Shrub	Y	N			X		
Baccharis halimifolia	Asteraceae	Perennial	Shrub/Tree	Y	N					X
Balduina angustifolia	Asteraceae	Annual/Biennial/Perennial	Forb/herb	Y	N					X
Baptisia lecontei	Fabaceae	Perennial	Forb/herb	Y	N			X		
Berlandiera subacaulis	Asteraceae	Perennial	Forb/herb	Y	Y		X	X	X	
Bidens alba	Asteraceae	Annual/Perennial	Forb/herb	Y	N			X	X	X
Boerhavia diffusa	Nyctaginaceae	Annual/Perennial	Forb/herb	Y	N				X	
Callicarpa americana	Lamiaceae	Perennial	Shrub	Y	N			X	X	
Cantinoa mutabilis	Lamiaceae	Perennial	Forb/herb	N	N	Gilchrist		X	X	X
Carphephorus sp.	Asteraceae	Perennial	Forb/herb	Y	NA	NA				X
Carphephorus corymbosus	Asteraceae	Perennial	Forb/herb	Y	N					X

Carphephorus paniculatus	Asteraceae	Perennial	Forb/herb	Y	N					X
Centella asiatica	Apiaceae	Perennial	Forb/herb/Subshrub	Y	N			X		
Chaerophyllum tainturieri	Apiaceae	Annual	Forb/herb	Y	N		X	X		
Chamaecrista fasciculata	Fabaceae	Annual	Forb/herb	Y	N				X	X
Chrysopsis mariana	Asteraceae	Perennial	Forb/herb	Y	N				X	X
Chrysopsis scabrella	Asteraceae	Annual	Forb/herb	Y	N	Gilchrist, Suwannee				X
Chrysopsis subulata	Asteraceae	Perennial	Forb/herb	Y	Y	Gilchrist				X
Clitoria mariana	Fabaceae	Perennial	Forb/herb/Vine	Y	N			X	X	
Cnidoscolus stimulosus	Euphorbiaceae	Perennial	Forb/herb	Y	N		X	X	X	X
Commelina erecta	Commelinaceae	Perennial	Forb/herb	Y	N			X	X	X
Condea verticillata	Lamiaceae	Perennial	Forb/herb/Subshrub	N	N	Gilchrist				X
Conyza canadensis	Asteraceae	Annual/Biennial	Forb/herb	Y	N				X	X
Coreopsis basalis	Asteraceae	Annual	Forb/herb	N	N			X	X	
Coreopsis lanceolata	Asteraceae	Perennial	Forb/herb	Y	N	Columbia, Gilchrist		X		X
Coreopsis leavenworthii	Asteraceae	Perennial	Forb/herb	Y	N			X	X	X
Corydalis micrantha	Papaveraceae	Annual	Forb/herb	Y	N		X			
Crataegus sp.	Rosaceae	Perennial	Shrub/Tree	Y	N	NA	X			
Crocanthemum corymbosum	Cistaceae	Perennial	Forb/herb/Subshrub	Y	N			X	X	X
Crocanthemum georgianum	Cistaceae	Perennial	Forb/herb/Subshrub	Y	N			X		
Croptilon divaricatum	Asteraceae	Annual	Forb/herb	Y	N					X
Crotalaria lanceolata	Fabaceae	Annual	Forb/herb	N	N					X
Crotalaria pallida	Fabaceae	Perennial	Forb/herb/Subshrub	N	N	Gilchrist				X
Crotalaria rotundifolia	Fabaceae	Perennial	Forb/herb	Y	N			X		
Crotalaria spectabilis	Fabaceae	Annual	Forb/herb	N	N					X

Croton argyranthemus	Euphorbiaceae	Perennial	Forb/herb/Subshrub	Y	N			X	X	
Croton glandulosus	Euphorbiaceae	Annual	Forb/herb/Subshrub	Y	N			X	X	X
Croton michauxii	Euphorbiaceae	Annual	Forb/herb	Y	N			X	X	X
Cyclospermum leptophyllum	Apiaceae	Annual	Forb/herb	N	N	Gilchrist		X		
Descurainia pinnata	Brassicaceae	Annual/Biennial/Perennial	Forb/herb	Y	N		X			
Desmodium strictum	Fabaceae	Perennial	Forb/herb	Y	N	Gilchrist				X
Desmodium triflorum	Fabaceae	Perennial	Forb/herb	N	N					X
Diodia virginiana	Rubiaceae	Annual/Perennial	Forb/herb/Subshrub	Y	N			X	X	X
Distimake dissectus	Convolvulaceae	Perennial	Forb/herb/Vine	Y	N	Gilchrist		X	X	X
Drosera capillaris	Droseraceae	Perennial	Forb/herb	Y	N			X		
Edrastima uniflora	Rubiaceae	Annual	Forb/herb/Subshrub	Y	N				X	X
Elephantopus elatus	Asteraceae	Perennial	Forb/herb	Y	N					X
Erechtites hieraciifolius	Asteraceae	Annual	Forb/herb	Y	N				X	
Erigeron quercifolius	Asteraceae	Annual	Forb/herb	Y	N			X		
Erigeron strigosus	Asteraceae	Annual/Biennial/Perennial	Forb/herb	Y	N		X	X	X	X
Eriogonum tomentosum	Polygonaceae	Perennial	Forb/herb	Y	N				X	
Eryngium aquaticum	Apiaceae	Biennial	Forb/herb	Y	N	Gilchrist		X		
Eryngium aromaticum	Apiaceae	Perennial	Forb/herb	Y	N				X	
Eryngium baldwinii	Apiaceae	Biennial/Perennial	Forb/herb	Y	N			X		
Euphorbia cyathophora	Euphorbiaceae	Annual/Perennial	Forb/herb	Y	N	Gilchrist		X	X	X
Euphorbia heterophylla	Euphorbiaceae	Annual/Perennial	Forb/herb	Y	N	Gilchrist			X	X
Euthamia caroliniana	Asteraceae	Perennial	Forb/herb	Y	N					X
Froelichia floridana	Amaranthaceae	Annual	Forb/herb	Y	N			X	X	X
Galactia mollis	Fabaceae	Perennial	Forb/herb/Vine	Y	N				X	

Galactia sp.	Fabaceae	Perennial	Forb/herb/Vine	Y	NA	NA		X	X	
Galium aparine	Rubiaceae	Annual	Forb/herb/Vine	Y	N		X			X
Galium tinctorium	Rubiaceae	Perennial	Forb/herb	Y	N	Gilchrist		X		X
Gelsemium sempervirens	Gelsemiaceae	Perennial	Shrub/Vine	Y	N		X			
Geobalanus oblongifolius	Chrysobalanaceae	Perennial	Shrub/Subshrub	Y	N			X		
Geranium carolinianum	Geraniaceae	Annual/Biennial	Forb/herb	Y	N		X	X		
Glandularia aristigera	Verbenaceae	Annual/Perennial	Forb/herb/Subshrub	N	N		X	X	X	
Helianthus angustifolius	Asteraceae	Perennial	Forb/herb	Y	N	Gilchrist				X
Heterotheca subaxillaris	Asteraceae	Annual	Forb/herb	Y	N					X
Hexasepalum teres	Rubiaceae	Annual/Perennial	Forb/herb	Y	N				X	X
Hieracium gronovii	Asteraceae	Perennial	Forb/herb	Y	N				X	
Hieracium megacephalon	Asteraceae	Perennial	Forb/herb	Y	N	Gilchrist	X	X		
Houstonia procumbens	Rubiaceae	Perennial	Forb/herb	Y	N		X			
Hydrocotyle umbellata	Araliaceae	Perennial	Forb/herb	Y	N			X	X	X
Hymenopappus scabiosaeus	Asteraceae	Biennial	Forb/herb	Y	N			X		
Hypericum gentianoides	Clusiaceae	Annual	Forb/herb	Y	N				X	X
Hypericum hypericoides	Clusiaceae	Perennial	Shrub/Subshrub	Y	N				X	X
Hypericum myrtifolium	Clusiaceae	Perennial	Shrub/Subshrub	Y	N			X		X
Hypericum tetrapetalum	Clusiaceae	Perennial	Shrub/Subshrub	Y	N			X	X	X
Ilex glabra	Aquifoliaceae	Perennial	Shrub	Y	N			X		
Indigofera hirsuta	Fabaceae	Annual	Forb/herb/Subshrub	N	N					X
Indigofera spicata	Fabaceae	Annual/Perennial	Forb/herb	N	N					X
Ipomoea hederacea	Convolvulaceae	Annual	Forb/herb/Vine	N	N	Gilchrist				X
Ipomoea hederifolia	Convolvulaceae	Annual	Forb/herb/Vine	Y	N				X	X

Ipomopsis rubra	Polemoniaceae	Biennial	Forb/herb	Y	N				X	
Juncus marginatus	Juncaceae	Perennial	Graminoid	Y	N				X	
Kalmia hirsuta	Ericaceae	Perennial	Shrub	Y	N				X	
Krigia cespitosa	Asteraceae	Annual	Forb/herb	Y	N					X
Lachnanthes caroliana	Haemodoraceae	Perennial	Forb/herb	Y	N				X	
Lachnocaulon beyrichianum	Eriocaulaceae	Perennial	Forb/herb	Y	N	Gilchrist	X	X	X	
Lactuca graminifolia	Asteraceae	Biennial/Perennial	Forb/herb	Y	N		X	X		
Lechea deckertii	Cistaceae	Perennial	Forb/herb/Subshrub	Y	N	Gilchrist, Suwannee				X
Lepidium virginicum	Brassicaceae	Annual/Biennial/Perennial	Forb/herb	Y	N		X	X		X
Liatris gracilis	Asteraceae	Perennial	Forb/herb	Y	N					X
Liatris sp.	Asteraceae	Perennial	Forb/herb	Y	NA	NA				X
Liatris tenuifolia	Asteraceae	Perennial	Forb/herb	Y	N	Columbia				X
Linum floridanum	Linaceae	Perennial	Forb/herb	Y	N	Gilchrist, Suwannee			X	X
Lonicera sempervirens	Caprifoliaceae	Perennial	Vine	Y	N	Gilchrist		X	X	
Lythrum alatum	Lythraceae	Perennial	Forb/herb/Subshrub	Y	N	Gilchrist			X	
Mecardonia procumbens	Plantaginaceae	Annual/Perennial	Forb/herb	Y	N	Levy			X	
Medicago lupulina	Fabaceae	Annual/Perennial	Forb/herb	N	N		X	X		
Melothria pendula	Cucurbitaceae	Perennial	Forb/herb/Vine	Y	N				X	X
Mimosa quadrivalvis	Fabaceae	Perennial	Forb/herb/Vine	Y	N	*		X		
Mimosa strigillosa	Fabaceae	Perennial	Forb/herb/Subshrub	Y	N			X		
Mollugo verticillata	Molluginaceae	Annual	Forb/herb	N	N	Gilchrist			X	
Monarda punctata	Lamiaceae	Annual/Biennial/Perennial	Forb/herb/Subshrub	Y	N	Suwannee				X
Oenothera biennis	Onagraceae	Biennial	Forb/herb	Y	N	Suwannee				X
Oenothera laciniata	Onagraceae	Annual/Perennial	Forb/herb	Y	N			X	X	

Oenothera simulans	Onagraceae	Annual	Forb/herb	Y	N			X	X	X
Oenothera speciosa	Onagraceae	Perennial	Forb/herb/Subshrub	N	N	Gilchrist			X	
Opuntia austrina	Cactaceae	Perennial	Shrub	Y	N	Columbia		X		
Oxalis corniculata	Oxalidaceae	Annual/Perennial	Forb/herb	Y	N		X	X	X	X
Packera glabella	Asteraceae	Annual	Forb/herb	Y	N		X			
Palafoxia integrifolia	Asteraceae	Annual	Forb/herb	Y	N					X
Paronychia patula	Caryophyllaceae	Annual	Forb/herb	Y	N					X
Paronychia rugelii	Caryophyllaceae	Annual	Forb/herb	Y	N	Columbia			X	X
Passiflora incarnata	Passifloraceae	Perennial	Forb/herb/Vine	Y	N				X	
Pectis prostrata	Asteraceae	Annual	Forb/herb	Y	N	Gilchrist		X		
Penstemon multiflorus	Plantaginaceae	Perennial	Forb/herb	Y	N			X	X	
Pentodon pentandrus	Rubiaceae	Annual	Forb/herb	Y	N	Gilchrist				X
Phlox drummondii	Polemoniaceae	Annual	Forb/herb	N	N		X	X	X	
Phlox floridana	Polemoniaceae	Perennial	Forb/herb	Y	N			X		
Phyla nodiflora	Verbenaceae	Perennial	Forb/herb	Y	N			X	X	
Physalis arenicola	Solanaceae	Perennial	Forb/herb	Y	N	Gilchrist	X	X		
Pinguicula pumila	Lentibulariaceae	Annual/Perennial	Forb/herb	Y	N		X			
Piriqueta cistoides	Turneraceae	Annual/Perennial	Forb/herb/Subshrub	Y	N		X	X	X	X
Pityopsis graminifolia	Asteraceae	Perennial	Forb/herb	Y	N					X
Plantago virginica	Plantaginaceae	Annual/Biennial	Forb/herb	Y	N		X			
Polygala incarnata	Polygalaceae	Annual	Forb/herb	Y	N	Gilchrist			X	
Polygala nana	Polygalaceae	Annual	Forb/herb	Y	N		X	X	X	X
Polygonum nesomii	Polygonaceae	Perennial	Subshrub	Y	Y	Columbia				X
Polygonum pinicola	Polygonaceae	Annual	Forb/herb/Subshrub	Y	N	Columbia				X

Polypremum procumbens	Tetrachondraceae	Annual/Perennial	Forb/herb	Y	N				X	X
Prunus serotina	Rosaceae	Perennial	Shrub/Tree	Y	N		X			
Prunus umbellata	Rosaceae	Perennial	Shrub/Tree	Y	N		X			
Pseudognaphalium	Asteraceae	Annual/Biennial	Forb/herb	Y	N					X
Pterocaulon pycnostachyum	Asteraceae	Perennial	Forb/herb/Subshrub	Y	N			X		X
Ptilimnium capillaceum	Apiaceae	Annual	Forb/herb	Y	N	Gilchrist	X	X		
Rhexia mariana	Melastomataceae	Perennial	Forb/herb	Y	N				X	
Rhus copallinum	Anacardiaceae	Perennial	Shrub/Tree	Y	N				X	
Rhynchosia difformis	Fabaceae	Perennial	Forb/herb/Vine	Y	N				X	
Rhynchosia michauxii	Fabaceae	Perennial	Vine	Y	N				X	
Rhynchosia minima	Fabaceae	Perennial	Forb/herb/Subshrub/Vine	Y	N	Gilchrist, Suwannee				X
Richardia brasiliensis	Rubiaceae	Annual/Perennial	Forb/herb	N	N	Gilchrist, Levy		X	X	X
Rubus cuneifolius	Rosaceae	Perennial	Subshrub	Y	N		X	X		
Rubus trivialis	Rosaceae	Perennial	Subshrub/Vine	Y	N		X			
Rudbeckia hirta	Asteraceae	Annual/Biennial/Perennial	Forb/herb	Y	N				X	
Rumex hastatulus	Polygonaceae	Perennial	Forb/herb	Y	N		X	X		
Sagittaria graminea	Alismataceae	Perennial	Forb/herb	Y	N	*				X
Sagittaria kurziana	Alismataceae	Perennial	Forb/herb	Y	N			X	X	X
Salvia lyrata	Lamiaceae	Perennial	Forb/herb	Y	N					X
Scoparia dulcis	Plantaginaceae	Annual/Perennial	Forb/herb/Subshrub	Y	N				X	X
Scutellaria integrifolia	Lamiaceae	Perennial	Forb/herb	Y	N				X	
Serenoa repens	Arecaceae	Perennial	Shrub/Tree	Y	N			X		
Sericocarpus tortifolius	Asteraceae	Perennial	Forb/herb	Y	N				X	
Sida rhombifolia	Malvaceae	Annual/Perennial	Forb/herb/Subshrub	Y	N	Gilchrist		X	X	

Sida ulmifolia	Malvaceae	Perennial	Forb/herb/Subshrub	Y	N				X	X
Smilax smallii	Smilacaceae	Perennial	Shrub/Vine	Y	N				X	
Solanum chenopodioides	Solanaceae	Perennial	Forb/herb	Y	N	Gilchrist		X		X
Solidago canadensis	Asteraceae	Perennial	Forb/herb	Y	N	Gilchrist, Suwannee				X
Sonchus asper	Asteraceae	Annual	Forb/herb	N	N			X		
Sophronanthe hispida	Plantaginaceae	Perennial	Forb/herb	Y	N			X	X	X
Spermacoce remota	Rubiaceae	Annual/Perennial	Forb/herb/Subshrub	Y	N	Gilchrist				X
Spermacoce verticillata	Rubiaceae	Perennial	Subshrub	N	N	Gilchrist		X	X	
Stachys floridana	Lamiaceae	Perennial	Forb/herb	Y	N			X	X	
Stillingia sylvatica	Euphorbiaceae	Perennial	Forb/herb	Y	N			X		
Stylisma patens	Convolvulaceae	Perennial	Forb/herb/Vine	Y	N	*		X	X	
Symphyotrichum walteri	Asteraceae	Perennial	Forb/herb	Y	N	Gilchrist				X
Tephrosia chrysophylla	Fabaceae	Perennial	Forb/herb	Y	N				X	
Tephrosia spicata	Fabaceae	Perennial	Forb/herb	Y	N	Gilchrist			X	
Thlaspi arvense	Brassicaceae	Annual	Forb/herb	N	N	Gilchrist	X			
Trichostema dichotomum	Lamiaceae	Annual	Forb/herb	Y	N					X
Trichostema setaceum	Lamiaceae	Annual	Forb/herb	Y	N	Suwannee				X
Utricularia subulata	Lentibulariaceae	Annual/Perennial	Forb/herb	Y	N	Gilchrist	X	X		
Vaccinium arboreum	Ericaceae	Perennial	Shrub/Tree	Y	N			X		
Verbena brasiliensis	Verbenaceae	Annual	Forb/herb/Subshrub	N	N			X	X	X
Verbena halei	Verbenaceae	Perennial	Forb/herb/Subshrub	Y	N	Gilchrist		X	X	X
Vernonia gigantea	Asteraceae	Perennial	Forb/herb	Y	N				X	X
Veronica arvensis	Plantaginaceae	Annual	Forb/herb	N	N		X			
Veronica peregrina	Plantaginaceae	Annual	Forb/herb	Y	N	Gilchrist			X	

Vicia sativa	Fabaceae	Annual	Forb/herb/Vine	N	N	Gilchrist	X			
Vitis cinerea	Vitaceae	Perennial	Vine	Y	N	Gilchrist		X		
Vitis rotundifolia	Vitaceae	Perennial	Vine	Y	N	Gilchrist		X		
Wahlenbergia marginata	Campanulaceae	Perennial	Forb/herb	N	N	Gilchrist		X	X	
Xyris fimbriata	Xyridaceae	Perennial	Forb/herb	Y	N	Levy		X	X	X
Unknown.G5.1	NA	NA	NA	NA	NA	NA				X
