# Supplementary material for: Integrated vegetation management within electrical transmission landscapes promotes floral resource and flower-visiting insect diversity
Source: PLoS One. 2024 Aug 21;19(8):e0308263. doi: 10.1371/journal.pone.0308263 (PMC11338444; doi:10.1371/journal.pone.0308263)
Supplement: S3 Table — Minimum, maximum, average (lsmean), standard error, and significance observed for abundance and richness for five flower-visiting insect groups across the three management treatments (i.e., high, mid, and low management). This table corresponds to Fig 4 in the manuscript. Letters indicating statistical differences based on post hoc tests assuming Sidak correction for multiple comparisons. Confidence level used: 0.95, significance level used: σ = 0.05 (a>b>c). (PDF) [file pone.0308263.s006.pdf]

Integrated vegetation management within electrical transmission landscapes promotes floral resource and flower-visiting insect diversity

Chase B. Kimmel[1*], Ivone de Bem Oliveira[1], Joshua W. Campbell[1,2], Emily Khazan[1], Jonathan S. Bremer[1,3], Kristin Rossetti[1], Matthew Standridge[1], Tyler J. Shaw[1], Samm Epstein[1], Alexandra Tsalickis[1,4], and Jaret C. Daniels[1,5]

[1] McGuire Center for Lepidoptera and Biodiversity, Florida Museum of Natural History, University of Florida, Gainesville, Florida, United States of America

[2] United States Department of Agriculture Agricultural Research Service Northern Plains Agricultural Research Laboratory, Sidney, Montana, United States of America

[3] Florida Department of Agriculture and Consumer Services, Division of Plant Industry, Entomology Section, Gainesville, Florida, United States of America

[4] Department of Geosciences, Auburn University, Auburn, Alabama, United States of America

[5] Department of Entomology and Nematology, University of Florida, Gainesville, Florida, United States of America

* Corresponding author
E-mail: cbkimmel@ufl.edu (CBK)

S3 Table. Parameter summary table for flower-visitor insect groups. Minimum, maximum, average (lsmean), standard error, and significance observed for abundance and richness for five flower-visiting insect groups across the three management treatments (i.e., high, mid, and low management). This table corresponds to Fig 4 in the manuscript. Letters indicating statistical differences based on post hoc tests assuming Sidak correction for multiple comparisons. Confidence level used: 0.95, significance level used: σ = 0.05 (a>b>c).

Parameter	Treatment	Flower-visitor Group	Min	Max	Lsmean (sig.*)	Standard Error
Abundance	High	Bee	5.02	36.88	12.56 (a)	0.46
	Mid		1.85	24.15	6.74 (b)	0.45
	Low		1.82	20.59	5.87 (b)	0.45
	High	Wasp	2.06	4.24	2.99 (a)	0.03
	Mid		1.10	2.37	1.63 (b)	0.03
	Low		0.77	1.88	1.19 (c)	0.03
	High	Beetle	1.36	15.24	6.25 (a)	0.24
	Mid		0.76	11.98	4.25 (b)	0.23
	Low		0.43	11.07	3.39 (c)	0.23
	High	Butterfly/Moth	0.25	2.73	1.21 (a)	0.04
	Mid		0.15	1.29	0.59 (b)	0.04
	Low		0.10	1.58	0.61 (b)	0.04
	High	Fly	0.15	22.80	3.91 (a)	0.34
	Mid		0.14	18.44	3.63 (a)	0.33
	Low		0.03	5.78	1.13 (b)	0.33
Richness	High	Bee	0.88	2.01	1.35 (a)	0.02
	Mid		0.87	1.80	1.22 (c)	0.01
	Low		0.94	1.97	1.29 (b)	0.01
	High	Wasp	0.48	3.37	1.60 (a)	0.03
	Mid		0.43	2.39	1.11 (b)	0.03
	Low		0.49	1.67	0.88 (c)	0.03
	High	Beetle	0.30	4.15	1.36 (a)	0.05
	Mid		0.40	3.11	1.26 (a)	0.05
	Low		0.36	2.46	1.08 (b)	0.05
	High	Butterfly/Moth	0.22	0.95	0.52 (a)	0.01
	Mid		0.18	0.74	0.41 (b)	0.01
	Low		0.16	0.67	0.38 (b)	0.01
	High	Fly	0.14	1.00	0.47 (a)	0.02
	Mid		0.13	0.90	0.42 (a)	0.02
	Low		0.10	0.70	0.32 (b)	0.02
